# The Micro-Pillar Shear-Stress Sensor MPS^3^ for Turbulent Flow

**DOI:** 10.3390/s90402222

**Published:** 2009-03-30

**Authors:** Sebastian Große, Wolfgang Schröder

**Affiliations:** 1 Institute of Aerodynamics, RWTH Aachen University, Wüllnerstraße 5a, 52062 Aachen, Germany; 2 Laboratory for Aero and Hydrodynamics, Delft University of Technology, 2628 CA Delft, The Netherlands E-Mail: office@aia.rwth-aachen.de

**Keywords:** Fluid Mechanics, Turbulence, Wall-Shear Stress, Skin Friction, Micro-Pillar Shear-Stress Sensor MPS^3^

## Abstract

Wall-shear stress results from the relative motion of a fluid over a body surface as a consequence of the no-slip condition of the fluid in the vicinity of the wall. To determine the two-dimensional wall-shear stress distribution is of utter importance in theoretical and applied turbulence research. In this article, characteristics of the Micro-Pillar Shear-Stress Sensor MPS^3^, which has been shown to offer the potential to measure the two-directional dynamic wall-shear stress distribution in turbulent flows, will be summarized. After a brief general description of the sensor concept, material characteristics, possible sensor-structure related error sources, various sensitivity and distinct sensor performance aspects will be addressed. Especially, pressure-sensitivity related aspects will be discussed. This discussion will serve as ‘design rules’ for possible new fields of applications of the sensor technology.

## 1. Introduction

The interaction of a fluid with a surface creates mechanical stresses, which can be divided into the wall-normal pressure *p_wall_* and the wall-shear stress τ*_wall_*, acting along the wall-parallel direction. In Newtonian fluids, the wall-shear stress is expressed by

(1) $$\tau_{\mathrm{wall}}=\eta {\frac{\partial U}{\partial y}|}_{\mathrm{wall}},$$

where η is the dynamic viscosity of the fluid and ∂*U*/∂*y* is the wall-normal gradient of the mean stream-wise velocity *U*(*y*) adjacent at the wall.

To determine the wall-shear stress is of utter importance in theoretical and applied turbulence research. The mean wall-shear stress defines the friction velocity $u_{\tau }=\sqrt{\tau_{\mathrm{wall}}/\rho }$, where ρ is the density of the fluid. The friction velocity is relevant to determine non-dimensional variables such as *u*^+^ or *y*^+^ and serves as an important reference to judge the quality of turbulence models. The accurate determination of wall friction would allow the more precise identification of scaling parameters and scaling laws, e.g., for the mean velocity field or for turbulent energy spectra.

The fluctuating wall-shear stress distribution represents a footprint of near-wall turbulent structures and its measurement gives insight into the turbulent momentum transfer processes in the proximity of the wall and is as such of fundamental importance for the basic understanding of turbulent processes.

Furthermore, the measurement of the skin friction is essential in many technical applications, e.g., in the field of drag reduction and performance enhancement for transportation vehicles, where the viscous surface drag plays a major role. In flow control applications, the assessment of the local wall-shear stress or of the wall-shear stress distribution is a necessary prerequisite for the formulation of low-dimensional control models.

First preliminary results of a micro-pillar sensor application have been described in [1], the static and dynamic calibration of micro-pillar sensors have been reported in [2] and [3], respectively, and successful applications of the Micro-Pillar Shear-Stress Sensor MPS^3^ have been discussed in [4–7]. The focus of the present article will be more general. The sensor characteristics will be discussed allowing the reader to judge the applicability of the sensor technique in new fields. It will become evident that the choice of an appropriate sensor is an intrinsic task due to the complexity of different constraints and it needs to be made with great care taking fluid mechanical restrictions and sensor-sensitivity based requirements as well as structure mechanical requisites into consideration.

The article is structured as follows. First, a brief general description of the sensor concept will be given in section 2.. The sensor manufacturing will be briefly addressed in section 3. before material characteristics are discussed in section 4.. Sensor-structure related errors and several sensitivity aspects and diverse sensor performances are discussed at length in sections 5., 6. and 7., respectively. This discussion will yields some kind of ‘design rules’ for possible new fields of applications of the sensor technology. Finally, a conclusion will wrap up the article.

## 2. General Description of the Micro-Pillar Shear-Stress Sensor MPS^3^

The Micro-Pillar Shear-Stress Sensor MPS^3^ is based on thin cylindrical structures, which bend due to the exerted fluid forces, and as such the technique belongs to the indirect group [8] of sensors since the wall-shear stress is derived from the relation between the detected velocity gradient in the viscous sublayer and the local surface friction. Several methods such as wall-wire measurements [9], diverse micro-cantilevers or the assessment of the wall-shear stress from near-wall micro-Particle-Image Velocimetry (*μ*PIV) measurements [10] have been proposed to indirectly measure the wall-shear stress by applying its relation to the near-wall velocity gradient. Static sublayer surface fences have been used to measure mean surface skin friction in turbulent flows, for which the shear stress is taken to be proportional to a pressure drop ∂*p*/∂*x_i_* across the fence [9, 11].

**Figure 1.**
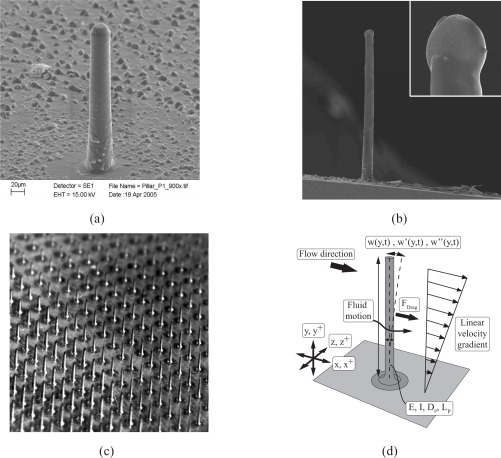
(*a*,*b*) Scanning-Electron Microscope (SEM) images of single pillars and (*c*) image of a pillar array. (*d*) Mechanical model of the pillar sensor.

The pillars are manufactured from the elastomer polydimethylsiloxane (PDMS, Dow Corning Sylgard 184) at diameters in the range of microns such that they are flexible and easily deflected by the fluid forces to ensure a high sensitivity of the sensor. Single pillars are shown in figures 1(a) and 1(b), acomplete micro-pillar array allowing the assessment of the spatial wall-shear stress distribution is illustrated in figure 1(c), and a schematic of the mechanical model is given in 1(d).

As a consequence of the limited region, in which the linear relation between near-wall velocity gradient and wall-shear stress applies, the sensor length *L_p_* is forced to be completely immersed within the viscous sublayer of the flow. Experimental and numerical results [12, 13] indicate that the velocity profile in the vicinity of the wall can be assumed linear up to *y*^+^ = 5÷6, where *y*^+^ = *yν*/*u*_τ_ is the non-dimensional wall-distance in viscous units with ν as the kinematic viscosity of the fluid and *u*_τ_ as the friction velocity. The kinematic viscosity of water is approximately 10^−6^ *m*^2^/*s*, that of air 1.5 × 10^−5^ *m*^2^/*s*. The friction velocity can be expressed as a function of bulk Reynolds number and thereby depends on the large-scale geometry of the flow field and the bulk velocity. Typical pillar lengths of sensors applied in the past measurements range in the order of 150÷700 *μm*. In liquid medium flow facilities with typical bulk-scale dimensions of 10^−2^÷10^−1^ *m* and typical values of the friction velocity in the order of 10^−2^ *m*/*s* this allows the assessment of wall-shear stress at Reynolds numbers up to *Re_b_* = 10^4^÷10^5^. In boundary layer facilities with air such as that described in [14, 15] with typical dimensions of 10^0^ *m* measurements at Reynolds numbers up to *Re*_Θ_ = 10^3^÷10^4^ could be performed with the aforementioned pillar length. Note that the size *L_p_* = 5 *l*^+^ should be considered already an upper limit to the possible pillar length. Due to the integration of the flow field along the pillar length it would be desirable to protrude as little as possible into the viscous sublayer. However, it goes without saying, that a shorter sensor structure also influences the sensor sensitivity and its static response.

The question how far the near-wall velocity field can be considered an adequate representative of the local mean and fluctuating wall-shear stress has been discussed in great detail in [2, 7]. Some further discussion can be found in section 6.1. of this paper. The authors concluded that the measurement of mean wall-shear stress and of its intensity by determining the velocity gradient in the vicinity of the wall is generally possible. That is, the mean velocity and the intensity of velocity fluctuations within the viscous sublayer can be assumed constant enough such that the corresponding wall-shear stress properties can be deduced from the integrative quantity measured by the micro-pillar shear-stress sensor. Nonetheless, it needs to be taken into account that due to the integrative character of the sensor with respect to the flow field along the wall-normal direction, any non-constant distribution of statistical turbulence characteristics along the sensor length can hardly be detected and consequently, values of such terms measured with the micro-pillar sensor should only carefully be treated a suitable direct representative of the corresponding wall-shear stress characteristics. Especially higher-order moments of the velocity fluctuations in the vicinity of the wall such as the skewness and the flatness show a non-constant distribution, which is why these wall-shear stress properties can most likely not be reliably determined by integrating the corresponding velocity fluctuation quantities. Note, the necessity of linear shear flow exerted on the structure, i.e., the complete immersion of the sensor posts within the viscous sublayer is further given since the sensor structure is statically calibrated in the linear shear flow of a plate-cone rheometer. That is, the load cases during calibration and measurement need to be identical such that calibration results can directly be used to quantitatively determine turbulent shear layer wall-shear stress.

The sensor structure has a minimum dimension in the wall-parallel plane thereby reducing the spatial averaging. For the range of the above mentioned Reynolds numbers the wall-parallel dimension of the sensor, i.e., its non-dimensionalized diameter *D_p_*^+^, in viscous units is *D_p_*^+^ ≤ 1, where *D_p_*^+^ = *u*_τ_*D_p_*/ν. The current manufacturing process, which will be further described in the following section, allows a wide range of possible geometric properties of the sensors leading to aspect ratios *L_p_*/*D_p_* of up to 15÷25. The dynamic calibration of micro-pillar sensor structures has evidenced the dynamic behavior of the wall-shear stress sensor in air to strongly differ from that in water [3]. That is, in liquids the sensor structures show low-pass filter characteristics, whereas a strong resonance due to the low damping in air is evident. It is needless to say that the low-pass filter characteristics are favorable especially if turbulent frequencies larger than the damped eigenfrequency of the structure are expected. However, when turbulent frequencies are reasonably lower than the damped eigenfrequency even a resonant structure can be used for the measurements. For both fluid media, the sensor has evidenced to possess a reasonably constant gain at frequencies below the eigenfrequency. It is needless to say that a large dynamic bandwidth of the mechanical components of the sensor would be desirable. On the other hand, the small detectable forces of the fluctuating wall-shear stress require a small stiffness of the sensor, which consequently results in a lower natural frequency and dynamic bandwidth of the sensor structure. To be more precise, the sensor properties need to be chosen respecting static and dynamic characteristics. It further needs to be taken into account that not only the dynamic response determines the ability of the sensor to detect the high-frequency fluctuations. Since the highest frequencies are generally associated with the smallest scales in turbulent flows, it is necessary that the sensor length is reasonably small compared to these structures since otherwise, the integration of small-scale structures along the sensor length would impede their detection. Note that only a few of the existing sensor designs presented in the literature have actually been calibrated. One of these sensors is a floating element shear-stress sensor reported by [16, 17]. These authors calibrated the sensor in a square duct using an acoustic plane-wave excitation. The plane wave was generated using a compression driver and the instantaneous wall-shear stress was derived from the acoustic pressure measured by a microphone installed opposite the shear-stress sensor. This technique, however, can only be applied for the calibration of wall-mounted sensors (e.g., thermal or floating element sensors). A further technique to dynamically calibrate near-wall hot-wires and hot-films is reported e.g. in [18, 19].

Besides the above mentioned aspects, which determine the maximum allowable sensor length, the pillar length also needs to be chosen reasonably small to consider the sensor structure non-intrusive for the flow field. In turbulent shear flows, it is generally accepted sufficient that if the sensor structures are fully immersed in the viscous sublayer no disturbances are caused outside the viscous sublayer, and hence, global changes of the flow field in the buffer and logarithmic region of the shear layer do not occur. Wall-shear stress statistics, turbulent spectra, and spatial two-point correlations calculated from measurements with the pillars installed in the streamwise direction allowed to confirm the low intrusiveness of the technique and no interaction of the sensor structures. To further corroborate the low intrusive interference of the sensor and to ensure a purely local effect on the flow field near the sensor structure the flow field around the pillars has been examined using *μ*PIV [20]. The flow-field studies have been performed in a plate-cone rheometer. Such devices generate a plane linear shear flow with constant shear rate over a sufficiently wide spatial region and velocity range such that the drag force distribution exerted on the sensor structure is identical to that in the viscous sublayer of a turbulent boundary layer. The results showed the flow past the pillar to be well in the Stokes regime for the typical range of Reynolds numbers *Re*_*D*_*p*__ ≤ 1. To be more precise, the flow is no longer affected in a region three pillar diameters downstream of the structure. The streaklines possess a symmetric curvature. No separation zone in the wake region of the pillar can be identified. In other words, the flow past the pillar is well in the Stokes-flow or Oseen-flow regime. Consequently, it can be assumed that the flow field is only locally disturbed in a zone of only a few pillar diameters around the structure. That is, in the case of arrays, if a sufficiently large spacing of the pillars has been chosen, no global effect of the presence of the pillars immersed in the viscous sublayer is expected. Generally, pillar spacings of approximately 1÷2 *L_p_*, i.e., 15 − 25 *D_p_* have been chosen. Note, there exist no additional constraints due to the placement of necessary secondary structure or data read-out devices and the impeding limitation in spatial resolution is only the aforementioned local disturbance of the flow field by the pillar structure and the interference of neighboring pillars. In combination with the optical detection principle this allows extremely high local resolutions of the planar wall-shear distribution of 4 ÷ 5 *l*^+^ at the range of Reynolds numbers in previous experiments, i.e., compatible to assess characteristic turbulent length scales.

The sensor concept allows the two-directional detection of the fluid forces, since the symmetric geometry has no preferred sensitivity direction and furthermore, the sensor does not suffer from cross-axis sensitivity. Thus, the micro-pillar sensor enables the measurement of the two wall-parallel components of the wall-shear stress.

Most of today’s representatives of wall-shear stress sensors are so called Micro-Electro-Mechanical Systems (MEMS) that transform the mechanical reaction of the sensor to the exerted forces into an electrical signal, i.e., a voltage, by capacitive, inductive, or resistive means. Although these techniques possess a couple of advantages compared to the method described in this work, the mechano-optical principle, on which the Micro-Pillar Shear-Stress Sensor MPS^3^ is based, outperforms in various other regards. Some of the pros and cons will be briefly discussed in the following.

Most other sensor techniques reported in the literature differ from the MPS^3^ sensor design by their need for diverse secondary structures implemented on the ground. This can be either electrical supply wiring or mechanical read-out devices. It has already been mentioned that due to the optical detection principle there is no need for further structure on the wall and as such the assessment of the two-dimensional wall-shear stress distribution at high spatial resolution as small as 5 *l*^+^ is possible.

The use of more viscous fluids (e.g., the fluid used in the oil-channel facility described by [21]) allows the assessment of wall-shear stress at even higher spatial resolution in viscous units using the present sensor dimensions. Additionally, the total number of pillars can be chosen reasonably high such that shear-stress evaluations with vector numbers in the order of standard PIV evaluations are possible.

Furthermore, the optical detection principle allows the simultaneous determination of all wall-shear stress components without suffering from cross-axis sensitivity, which has been experienced by many other multi-directional devices.

On the other hand, the optical detection requires optical access, which can not in all cases be guaranteed, and limits the sensor at the current state to laboratory applications. Using conventional digital cameras limits the data by the available amount of memory. At full frame size, the number of images recordable at high recording frequency (recent commercially available high-speed cameras offer on-board memory of up to 12 *GByte*) is generally limited to a few thousand samples and as such a proper statistical evaluation of the data can not easily be performed. However, by reducing the sensor region, several 10000 to 100000 samples can be captured allowing for a reliable statistical treatment of the data.

The raw images require a very time-consuming image evaluation before the actual wall-shear stress data is obtained, whereas MEMS devices can output the wall-shear stress or a direct representative, e.g., an electrical voltage, almost immediately. This limits the use of the pillar sensor in flow control application, which would require a real-time evaluation of the data.

Let us summarize the aforementioned sensor properties. Depending on the geometrical properties of the sensor, the detection of characteristic scales of turbulent flow is possible mostly at low to moderate Reynolds numbers. Under certain circumstances, measurements even at high Reynolds numbers can be performed [6, 22]. Turbulent length scales in the order of 50 *μ*m and time scales in the order of a few *kHz* can be resolved. To find an optimum geometry of the pillar, fluid mechanical restrictions, sensor sensitivity based requirements and structure-mechanical considerations need to be addressed. These requirements are partly controversial and a compromise for the sensor properties must be found. Section 7. will discuss the sensor performance characteristics in more detail and will give the reader some ‘design rules’ for the layout of a proper sensor geometry.

## 3. Sensor Manufacture

**Figure 2.**
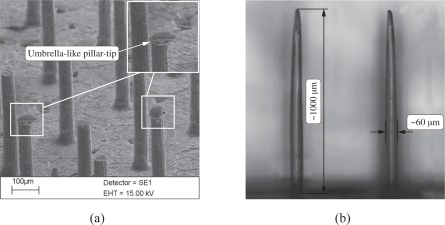
(*a*) Umbrella-like air inclusion during casting due to insufficient evacuation during fabrication and remaining gaps between wax mold and carrier substrate. (*b*) Typical pillar geometry mold in a blind hole evidencing a reduced diameter close to the pillar tip.

**Figure 3.**
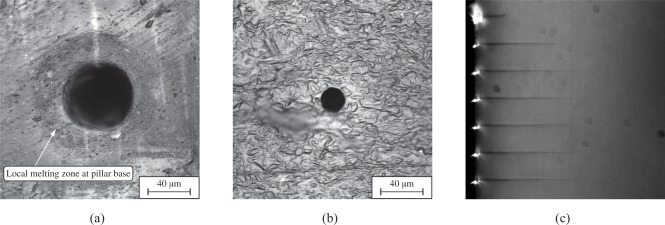
View of perforations into a wax foil at the entry and exit side of the laser beam. The entry diameter of the perforation is *D* = 38 *μm* (*a*) and corresponding exit hole with *D* = 15 *μm* in (*b*), respectively. (*c*) Side view of the perforations in the wax. Tapped blind holes (laser from left) for different number of laser pulses.

To produce filigree structures such as micro-pillars from PDMS elastomer at aspect ratios *L_p_*/*D_p_* as large as 15÷25 and at dimensions *L_p_* as small as 50÷1000 *μm*, replication processes of the sensors from a master are less intricate and expensive compared to a direct manufacturing of the sensor as a master. The procedure requires in a first step the manufacture of an appropriate mold, in which the elastomer is cured. The molds are made from wax allowing to fabricate sensor structures in a lost-mold process. The perforation of the wax with holes of diameters 10÷100 *μm* at high cylindricity is performed by locally sublimating the wax using laser irradiation. An excimer laser (Lambda Physik LPX140i laser) operating at 193 *nm* (ArF) has been used. The procedure allows to fabricate through borings or tapped blind holes. The former provide a precise perforation lengths defined by the wax material thickness. Although this technique also enables the manufacturing of pillars with a very constant diameter *D_p_* along the complete length, the risk of air trapped at the pillar tips cannot completely be avoided (figure 2(a)). In the case of tapped blind holes the energy distribution during perforation causes a slightly tapered contour of the pillar (figures 2(b) and 3(c)). The hole depth, i.e., the pillar length can be varied by the number of laser pulses. Depending on the desired length, the number of pulses ranges from 100 to 2000. A nearly linear relation between sensor length and pulse number has been determined. The pulse frequency showed to have little influence on the resulting structure, only at extremely high repetition frequencies, the local energy input was too high and caused the wax material to completely melt. Therefore, pulse frequency of only 50÷200 *Hz* have been applied. Even at lower frequencies, the wax mold indicates the existence of a local surface melting zone around the entry hole (figure 3(a)), causing the sensor posts to possess a smooth curvature at the base. The size and strength of the resulting curvature could be slightly influenced by the laser energy, the focus position and the frequency of laser pulses. It is needless to say that the curvature at the pillar mount has a major effect on the deflection of the pillar, however, this effect is accounted for when experimentally calibrating the structure. However, if the static or dynamic responses are assessed analytically, the local curvature at the base needs to be accounted for. At the exits, no similar effect could be observed.

Depending on the aperture used during the laser perforation the hole diameter can be varied from a few ten up to several hundred microns. As mentioned above, since the focus of the laser beam is not moved during the perforation process, holes with a trombone-like shape have been perforated resulting in a ratio between the exit and entry diameter of almost 0.35 ÷ 0.4 (figures 3(a) and 3(b)) during first feasibility studies at a wax thickness of 570 *μm*. An optimization of the focal position in relation to the wax surface and the manufacturing of shorter pillars increased the resulting ratio between exit and entry diameter to 0.75 ÷ 0.85 and even higher values.

The entry and exit holes have been inspected manually by microscopy evidencing a high degree of cylindricity. SEM images of final micro-pillar posts confirmed the these findings, however, no statistical evaluation has been done due to the insufficient number of sensors investigated by SEM.

In a second step, the PDMS elastomer is cast into the mold and cured. Both processes are performed under vacuum. Subsequently, the master mold is removed from the cured elastomer structure. Partly the removal by peel-off is possible, however, only at aspect ratios in the order of 10÷15, thereby allowing to keep the mold intact. At larger aspect ratios, the master mold was removed by wash-off.

To allow for a better optical detection of the micro-pillar, in a final step highly reflective hollow spheres are attached to the pillar tip. Further details of the manufacturing are described in great detail in [7].

## 4. Material

The micro-pillar sensors are manufactured from Dow Corning’s two-component silicone elastomer Sylgard^®^184, which belongs to the group of polydimethylsiloxanes (PDMS). Due to its mechanical, chemical, and optical properties, PDMS has become widely spread as material for nano- and microfluidic devices and for micro-structural mechanical sensor devices such as tactile [23], pressure [24] or acceleration sensors [25–27].

PDMS possesses a specific gravity of 1050 *kg*/*m*^3^, a tensile strength of 6.2 *MPa* and can reversibly be elongated up to 100% [28]. Note, PDMS is known to reach its final curing state not before seven days after molding. To ensure constant material properties, the sensors and the mechanical specimens for material studies should not be used for at least seven days after curing.

Its water absorption is less than 0.1% after seven days of immersion such that mechanical properties can be expected to not be influenced by a sensor being positioned in water flow facilities. The brittle point of the material is low with −65°*C* [28], ensuring measurements at +20°*C* not to suffer from glasifying effects and to be well situated on the rubbery plateau of the material. The material can further be applied at up to 100°*C* without experiencing any deterioration [28], however, the temperature dependence of the mechanical properties needs to be accounted for.

### 4.1. Young’s Modulus E

Young’s modulus (*E*) of PDMS can be tuned by up to a factor of 10, depending on the ratio of silicone and curing-agent and on the temperature cycle during curing (e.g. [29]). To determine Young’s modulus a tensile specimen has statically been extended such that the modulus can be calculated from the stress-strain relation. The advantage of this procedure is the possibility to identify the distribution of Young’s modulus as a function of stress. Young’s modulus has been calculated from the linear fit to the measured stress-strain relation. Due to the lateral contraction of the material, the non-dilated cross section *A*_0_ of the test specimen decreases by approximately 11% at elongations of ɛ = 0.20÷0.25. This has been accounted for in the calculation of Young’s modulus. The results for different curing cycles at a constant silicone to curing-agent ratio of 10÷1 revealed Young’s modulus to vary between 0.5÷2.0 *MPa*.

This sensitivity of Young’s modulus on the curing cycle temperature strongly affects the correct determination of the parameter and tests revealed Young’s modulus to differ by up to 30% between probes made of the same charge of silicone and under assumedly identical curing conditions. As such, it is critical to transfer Young’s modulus from a specimen to the actual pillar structure, if the curing procedure has not meticulously been identical. In consequence, this makes a static calibration of the sensor structures necessary, since Young’s modulus can not be determined from a specimen of the same material at a sufficient degree of accuracy. Furthermore, the determination of Young’s modulus directly from tests of the micro-pillar by means of static deflection to a known force or dynamic excitation, assuming it as a clamped cantilever, is not possible, because of a large uncertainty in the exact determination of the pillar’s geometry, and because of the intricacy and reliability of micro-structure mechanical tests.

To test the influence of different ambient fluids Young’s modulus of a probe has been evaluated after the pillar was immersed in water and water/glycerine mixtures for four hours up to seven days. The results indicated Young’s modulus not to be affected by this treatment, that is, Young’s modulus can be assumed constant under the impact of these fluids.

### 4.2. Hysteresis and Dissipation Factor

**Figure 4.**
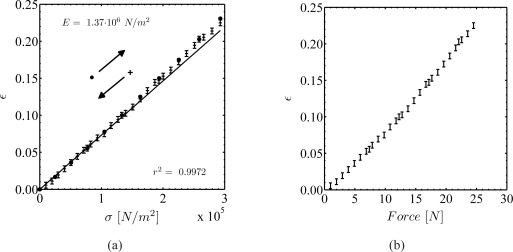
(*a*) Stress-strain relation for the tensile specimen, (*b*) force-strain relation for the tensile specimen.

Hysteresis describes the continuation of an effect after omission of its cause, e.g., the path-dependence of the reaction of a mechanical system to an oscillating force. To test the hysteresis of PDMS, load-unload cycles at strain rates in the range of 4÷30%/*min* have been performed. Figure 4(a) shows an exemplary result of the stress-strain relation for a tensile specimen, figure 4(b) the corresponding force-strain plot. The graph in figure 4(a) shows exemplary values recorded during load and unload cycles at a comparably low strain rate and the result clearly indicates the material to possess no or a negligible hysteresis. Similar findings could be observed for all strain rates during the experiments. This finding is in good agreement with the findings reported by [24] for photo-curable PDMS. However, these authors evidenced a strong degradation and a material-aging effect approximately 21 days after curing. This result could not be confirmed in the present study, which has been performed up to 28 days after curing using Dow Corning’s Sylgard®184 PDMS.

Furthermore, the loss tangent *tan* δ, which is a measure of the degree of dissipation of a mechanical mode, such as an oscillation, has been reported to be extremely low at values of *tan δ* ≤ 4·10^−3^ [27], indicating the material to possess a very low creep and drift [24], making PDMS ideal for dynamic measurements and for long-term wall-shear stress investigations.

### 4.3. Temperature Dependence of Material Characteristics

The results reported by [26] show the shear modulus *G* to vary slightly with temperature. In the temperature range *T* = 0÷100°*C* the authors evidenced *G* to linearly increase with 1.1 *kPa*/°*C*. Consequently, Young’s modulus *E*, which is related to *G* by *E* = 2(1+ν)·*G*, where ν is the Poisson ratio of the material, will also experience a temperature dependence. Note, in the case of rubber elastic materials, the Poisson ratio ν is approximately 0.5.

The linear and volume coefficient of thermal expansion are 3.0·10^−4^ %/°*C*, and 9.6·10^−4^ %/°*C*, respectively. The effect of the material temperature dependence on the measurement accuracy will be further discussed in the following section.

## 5. Sensor-Structure Related Errors Sources

This section discusses possible error sources related to the sensor structure itself. Measurement errors and the achievable accuracy of the optical acquisition principle have in detail been discussed in [2].

### 5.1. Sensor Misalignment

To discuss the different aspects of possible sensor misalignments, first the procedure of the sensor positioning in turbulent flow fields will be shortly elucidated. The actual micro-pillar sensor posts and the surface, on which they are mounted, are manufactured in one single step. This sensor ‘chip’ can be either directly flush-mounted in suitable grooves of a flow facility wall or in wall adapters, which can be further placed in the wall of the flow facility wall.

In the studies reported in [2, 4, 5] the sensor is mounted on an adapter that is placed in the calibration device as well as in the flow facility such that errors resulting from different sensor positions during calibration and measurement can be prevented. While the manufacturing process allows to fabricate sensor posts with a maximum deviation in orientation from the direction perpendicular to the surface of 0.05° and as such at an extremely low level of asymmetry, the last step, i.e., the mounting of the sensor ‘chip’ in the flow facility wall, can generally cause non-negligible misalignment errors. On the one hand, a parallel offset of the sensor ‘chip’ due to an imperfect flushness of the sensor chip and the surrounding wall (protrusion or recession) or on the other hand, a non-parallel orientation of the sensor ‘chip’ and the surrounding surface causing a one-sided vertical misalignment are possible. However, since the borders of the sensor mounts can be chosen reasonably far from the actual sensor post position (≥5 *L_p_*), local flow field disturbances caused by sensor-mount misalignments will not affect the sensor functioning.

The positioning of the sensor mount can be performed using micro-manipulating devices and visual inspection at microscopic magnification allows the detection of maximum vertical offsets of the sensor mount of less than 5 *μm*. Performing calibration and the wall-shear stress measurements with identical setups, any misalignment can be accounted for. Offsets in the order of less than 5 *μm* correspond to 0.1 *l*^+^ at typical Reynolds numbers in the experiments performed up to now such that no global flow field disturbance is expected and furthermore, the flow on the sensor ‘chip’ can be considered to be non-affected by the existence of vertical offsets of this size. As such, errors due to a misalignment of the structure can be considered negligible.

However, if measurements at higher Reynolds numbers, i.e., smaller absolute geometric dimensions of *l*^+^ are performed, even higher accuracy needs to be achieved. This could for example be managed by directly manufacturing the complete sensor on the flow facility wall without having the need to manually position sensor ‘chips’ on the wall or in wall adapters.

### 5.2. Aging and Altering Effects

It goes without saying that any change in structure-mechanically relevant sensor parameters and particularly Young’s modulus will modify the sensors sensitivity. The long-term (30 days-180 days) and short-term (30 min-7 days) repeatability tests at fluctuating and constant load evidenced an excellent agreement of the mean detected pillar deflections within ±2÷3% in all flow media used in the present studies, hence glycerine, water and air, i.e., no material aging was observed. Note, the sensor was stored in air between the tests. However, sensor calibrations have always been performed prior to measurements to ensure that any kind of sensor degradation or aging is accounted for.

Furthermore, changes of the sensor material due to long-term exposure to different environmental influences could lead to errors. Effects due to water absorption are negligible following the manufacturer’s information [28] and as mentioned above results from measurements of Young’s modulus of a tensile specimen with the material immersed in water and glycerine for up to seven days confirmed a negligible effect within the measurement accuracy.

### 5.3. Yielding-Induced Drift

It is well known that elastic materials tend to yield under constant stress causing the sensitivity of the sensor to change with time. It can be expected that altering mechanical properties and yielding would influence the mean detected wall-shear stress. To check for a possible drift of the sensor due to mechanical yielding, long-term (up to 30 *min*) measurements at different levels of sensor deflection and at constant and dynamic wall-shear stress under steady ambient conditions have been performed. Linear regressions of the normalized streamwise sensor deflection *w*(*L_p_*)/*w̄*(*L_p_*), where *w̄*(*L_p_*) is here considered the temporal mean pillar-tip deflection during the tests, possessed gradients of ±1.0%, i.e., a negligible drift. Note, yielding would have caused a clear positive tendency of the gradient. This has not been observed. It can further be concluded from these tests, that the sensor stays at a constant temperature and heating of the structure does not occur. it can be confidently assumed that any sensor heating due to internal frictional heating would be sufficient convected by the flow. A heating of the structure would have caused the sensor deflection to change in time.

### 5.4. Calibration-Related Aspects

It would be desirable to calibrate the micro-pillar structure in-situ, that is, to position the sensor for static calibration directly in the turbulent flow field. This would allow to avoid measurement errors arising from variances in the flow conditions during the rheometer calibration and the actual flow case, e.g., different temperatures, strongly differing Reynolds numbers *Re*_*D*_*p*__ determining the local flow field around the sensor structure, or from a possible sensor misalignments.

To obtain the relation between pillar deflection and wall-shear stress in the flow facility, it is necessary to quantitatively know the mean wall-shear stress at a high enough accuracy in the turbulent flow field, in which the calibration is performed. This requires on the one hand, the simultaneous application of an already calibrated device to assess the wall-shear stress or on the other hand, the wall-shear stress to be determined from an analytical relation assuming it to be valid to within the desired accuracy.

A problem of the in-situ calibration is the non-linearity of the static sensor response at low or high deflections. The velocity field in turbulent shear flows in the vicinity of the wall and hence, the wall-shear stress, are known to fluctuate strongly around their mean values. Simply assuming the arithmetic mean of the measured sensor deflections to be a linear-proportional representative of the mean wall-shear stress would yield an error especially at low deflections due to the non-linearity of the static response. Hence, an approach similar to that used for calibrations of hot-films or hot-wires - in case they are calibrated in highly fluctuating flow fields - is necessary. In [30] and [12], the use of higher-order polynomials for the calibration of hot-films is suggested. A similar procedure could also be used for the micro-pillar shear-stress sensor.

## 6. Sensitivity Aspects

Commonly, sensors are not only sensitive to one form of excitation. That is, a sensor response, e.g., in the case of the micro-pillar its deflection, not solely originates from the wall-shear stress, i.e., from the drag forces of the local small-scale flow field around the structure, but it is rather the consequence of several contributing effects. To judge the possibility of such a multi-sensitivity, the influence of secondary contributions will be discussed in the following.

Two different kinds of sensitivity-related aspects are possible. On the one hand, the direct impact of forces other than the drag force resulting from the fluid field surrounding the sensor structure, which we relate to the wall-shear stress, needs to be accounted for. As external forces, accelerations (e.g. due to accelerated flow facilities or test structures, in/on which the sensor is installed) and pressure forces need to be addressed. On the other hand, changes in the sensor sensitivity itself might have an deteriorating effect on the sensor function. Only temperature-related effects will be discussed in this section, since chemical and load-related changes of the sensor material and its mechanical properties have already been discussed in the preceding section and showed to have negligible effects on the sensor sensitivity.

### 6.1. Sensitivity to Pressure Gradients or Pressure Fluctuations

Most recent floating-element wall-shear stress sensors suffer from a certain degree of sensitivity to pressure forces. On the one hand, slightly differing pressure forces in pressure-driven flows act on the trailing and leading edges of floating-element sensors, and on the other hand, a pressure gradient between the sensor surface and the gap between the sensor and the substrate might be present, resulting in a wall-normal force, which contributes to the total load acting on the tethering springs. Similarly, it is possible that pressure forces act on the pillar structure (figure 7(a)) and in the following possible pressure force contributions will be addressed.

**Figure 5.**
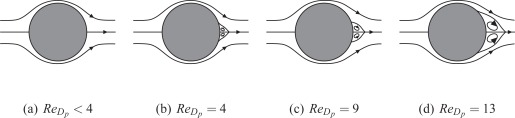
Two-dimensional flow field around a circular obstacle at different Reynolds number *Re*_*D*_*p*__ based on the diameter *D_p_* and the local velocity *U*. At *Re*_*D*_*p*__ ≥4 the flow detaches.

If the Reynolds number *Re*_*D*_*p*__ reaches a certain level, Stokes or Oseen flow around the structure can no longer be assumed and a detachment of the flow field at the lee-site of the pillar (figures 5(b) to 5(d)) causes additional differential pressure force contributions on the sensor structure. Since typical Reynolds numbers are *Re*_*D*_*p*__ ≤ 1, the Stokes condition can be assumed valid such that the flow field should symmetrically follow the pillar contour allowing to determine the total drag forces exerted by the local flow field around the sensor structure by analytical estimates given e.g. in [31], [32], or [33].

#### Mean Streamwise Pressure Gradient in Pressure-Driven Boundary Layers

In pressure-gradient driven flows, the mean pressure gradient Δ*p̄* along the streamwise extension of the sensor structure leads to a net pressure force. In the following the contribution of these global flow field pressure forces and the drag forces resulting from the local Stokes flow around the structure will be approximated for the typical range of Reynolds numbers of interest and it will be shown that pressure-gradient forces contributing to the total pillar deflection can be neglected.

It has been shown in [2] that the drag force exerted on the pillar per unit length by the local flow field can be calculated using the Oseen approximation for the drag load per unit length of a cylinder [32]

(2) qP(y)≈4πη2−loge(ReDp(y))⋅U(y).

The assumption of Oseen flow around the sensor structure is valid if the Reynolds number *Re*_*D*_*p*__= *U*(*L_p_*)*D_p_*/ν defined by the pillar diameter *D_p_* and the maximum velocity *U*(*L_p_*) at the pillar tip *y* = *L_p_* is *Re*_*D*_*p*__ ≤ 1.

The pressure forces per unit length *p_P_*(*y*) resulting from the mean streamwise pressure gradient ∂*p̄*/∂*x*, which act on the sensor structure can roughly be determined by

(3) $$p_{P}(y)\approx \Delta p\cdot D_{p},$$

where Δ*p* is the pressure drop along the pillar streamwise dimension, i.e., *D_p_*, and, hence, Δ*p*≈∂*p̄*/∂*x*·*D_p_*. Typical values of the local velocities *U*(*y*) at the present experimental conditions are of order 10^−1^ *m*/*s*, the Reynolds number *Re*_*D*_*p*__ is of order 10^−1^, and thereby the shear load per unit length *q_P_*(*y*) becomes 10^−4^÷10^−3^ *N*/*m* for water with a dynamic viscosity η being of order 10^−3^ *Pas*. With pillar diameters *D_p_* of 10^−5^÷10^−4^ *m* and a pressure drop Δ*p* along the pillar streamwise dimension, i.e., *D_p_*, of 10^−4^÷10^−3^ *N*/*m*^2^, the resulting pressure force per unit length *p*(*y*) becomes of order 10^−7^ *N*/*m* and as such, the ratio between the contributions *p_P_*(*y*)/*q_P_*(*y*) is of order 10^−4^÷10^−3^, and hence, can be considered negligible. Equations 2 and 3 allow to more generally calculate the ratio of shear and streamwise pressure gradient contributions to the pillar load *q_P_*(*y*)/*p_P_*(*y*), which should be of order 10^−2^.

#### Mean Wall-Normal Pressure Gradient in Turbulent Shear Layers

In the following the influence of mean wall-normal pressure gradients ∂*p̄*/∂*y* will be investigated. The non-dimensional wall-normal momentum equation (to first order) in the sublayer reads

(4) $$\frac{\partial \bar{p}}{\partial y}\approx -\rho \cdot \frac{\partial \bar{{\nu^{\prime }}^{2}}}{\partial y}=-{\rho u}_{\tau }{}^{3}/\nu \frac{\partial \nu^{+2}}{\partial y^{+}}\approx -{\rho u}_{\tau }{}^{3}/\nu \cdot 4\beta_{2}{}^{2}y^{+3},$$

where β_2_ is parameter defining the curvature of the wall-normal velocity profile, which will be discussed in further detail later in this section. The second part in equation 4 has been obtained applying equation 8. Typical dimensions, over which this wall-normal pressure acts, can be considered to be approximately *D_p_*, and, hence, the resulting wall-normal pressure Δ*p* exerted on the pillar is approximately ∂*p̄*/∂*y*·*D_p_*. The ratio *p_P_*(*y*)/*q_P_*(*y*) resulting from pressure forces due to the mean wall-normal pressure gradient and the drag forces reads

(5) $$\frac{p_{P}(y)}{q_{P}(y)}\approx \frac{4}{10\pi }\frac{u\tau^{2}}{\nu^{2}}\beta_{2}{}^{2}\Delta y^{+3}D_{p}{}^{2},$$

At the present experimental conditions *p_P_*(*y*)/*q_P_*(*y*) ≤ 0.02. Note, however, that the wall-normal pressure forces act differently than drag forces to the pillar bending. That is, wall-normal pressure forces can only contribute to the total sensor load in a deflected state of the pillar, whereas in the non-deflected state, they only act as a longitudinal force. In the deflected state, the integral of the wall-oriented pressure forces would yield further deflection, whereas that of pressure forces pointing away from the wall tend to restore the structure into its straight position. An FEM analysis of sensor geometries at deflections of *w*(*L_p_*)/*L_p_*≈0.1÷0.2 accounting for the drag load and the aforementioned pressure force acting concentrated at the sensor tip at similar ratios as discussed above indicated a negligible influence of the wall-normal pressure forces resulting from the mean pressure gradient to the total pillar-tip bending of ≈±0.50%.

#### Pressure Fluctuations in Turbulent Flows

In turbulent flows, fluctuating pressure forces resulting from turbulent fluid motion, will exert on the sensor structure. Such pressure fluctuations *p*^′^ can, for example, arise from acoustic pressure waves in gases or from turbulent momentum transfer, i.e., result from velocity fluctuations. Second, turbulent fluctuations cause local pressure fluctuations, which could exert on the sensor structure. Both possible contributions will be discussed in the following.

Similar to the considerations in [34], the impact of acoustic pressure fluctuations will be discussed here. Only pressure fluctuations *p*^′^ smaller than the pillar dimension will effectively impose pressure forces on the structure. It is reasonable to assume that the pillar diameter *D_p_* is the relevant dimension, at which wall-parallel pressure gradients induced by acoustic waves or turbulent motions can contribute to the deflection of the structure. For larger dimensions of the pressure fluctuations, the effect of the pressure fluctuations will be felt uniformly across the element. Characteristic wavelengths corresponding to the smallest length scales of pressure fluctuations can be estimated to be in the order of the wavelength of an acoustic pressure wave or of the smallest scales of eddies in turbulent flows. Acoustic pressure waves have a wavelength depending on the speed of sound in air *c* (340 *m*/*s* at room-temperature) and on the acoustic frequency *f*. The acoustic wavelength λ*_a_* is defined as λ*_a_* = *c*/ *f*. For pillar diameters in the order of 10÷50 *μm*, this corresponds to frequencies of approximately 6.8 *MHz*, a frequency higher than any frequency expected to occur in the turbulent shear flows of interest for the field of application of the sensor.

At the Reynolds numbers, at which the sensor is applied, the pillar diameter *D_p_* is less than 1 *l*^+^ and as such, pressure fluctuations induced by turbulent structures can be expected to not significantly contribute to the wall-parallel pillar load. However, to study the impact of turbulence induced pressure fluctuations *p*^′^ ≡ ∂*p*/∂*x_i_* on the total pillar bending in some more detail, the following considerations are helpful. A detailed review on the statistics associated with pressure fluctuations in turbulent flow at Reynolds numbers similar those in the experiments is given in [35]. Some further insight especially on the turbulent pressure fluctuations in the vicinity of the wall can be found in [36, 37].

First, the magnitude of pressure fluctuations need to be assessed. From the Navier-Stokes equations the rms of local pressure gradients can be expressed by (see e.g. [37, 38])

(6) $$\sqrt{\bar{{\left(\frac{\partial p}{{\partial x}_{i}}\right)}^{2}}}\approx 2\rho \nu \sqrt{\bar{{\left(\frac{\partial^{2}u_{i}}{{\partial x}_{j}{}^{2}}\right)}^{2}}}=2\rho u_{\tau }{}^{3}/\nu \sqrt{\bar{{\left(\frac{\partial^{2}u_{i}{}^{+}}{\partial x_{j}{}^{+2}}\right)}^{2}}}$$

The factor of 2 in this equation is representative for the level of fluctuations at *y*^+^ = 10 and rather overestimates the pressure fluctuations in the viscous sublayer. The second part of equation 6 is simply the non-dimensionalization using viscous scales, which helps understanding the following discussion. Writing a Taylor series expansion for the streamwise and wall-normal velocities yields

(7) $$u^{+}\left(y^{+}\right)=\alpha_{1}\cdot y^{+}+\alpha_{2}\cdot y^{+2}+\ldots$$

(8) $$v^{+}\left(y^{+}\right)=\beta_{1}\cdot y^{+}+\beta_{2}\cdot y^{+2}+\ldots$$

with β_1_ being essentially 0. From equation 6 the rms-values of local pressure gradients in the viscous sublayer read

(9) $$\frac{{\partial p}^{+}}{{\partial x}^{+}}=2\frac{\partial^{2}u^{+}}{\partial y^{+2}}\approx {4\alpha }_{2}$$

(10) $$\frac{\partial p^{+}}{\partial y^{+}}=2\frac{\partial^{2}v^{+}}{\partial y^{+2}}\approx 4\beta_{2}$$

**Figure 6.**
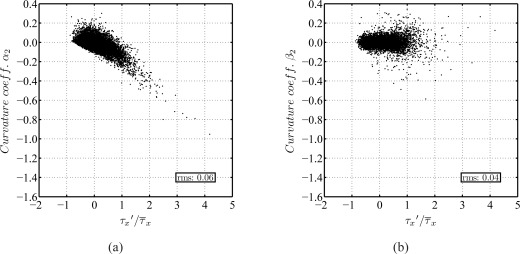
(*a*) Curvature coefficient α_2_ of the non-dimensionalized streamwise velocity profile *u*^+^(*y*^+^). (*b*) Curvature coefficient β_2_ of the non-dimensionalized profile *v*^+^(*y*^+^) of wall-normal velocities.

From DNS results of turbulent channel flow at Reynolds numbers similar to those in the present experiments [39], the value of α_2_ in equation 9 has been determined to be α_2_ ≈ 0.06. Note, this value represents the curvature of the velocity profile through the entire viscous sublayer, or, to be more precise its rms value (around zero). It has already been discussed that the mean velocity profile can be assumed linear, but instantaneous velocity profiles apparently possess a slight curvature, hence a value of α_2_ different from zero. The value of β_2_ is approximately 0.04 (rms around zero). Figure 6 shows the distribution of α_2_ and β_2_ as a function of τ^′^ /τ̄, respectively. While α_2_ appears to slightly depend on the value of τ^′^ /τ̄,it is evident that the magnitude of β_2_ does not increase. This suggests that the curvature in the streamwise velocity profile becomes a problem before the wall-normal pressure gradient does.

#### Wall-Parallel Pressure Fluctuations

**Figure 7.**
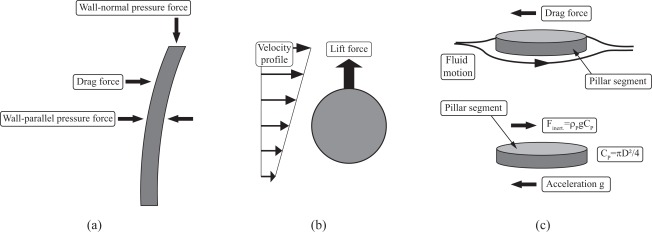
(*a*) Overview of possible pressure and Stokes/Oseen drag force contributions. (*b*) Lift force induced by a shear flow over a segment of the micro-pillar sensor. (*c*) Effect of fluid induced drag and inertial forces on a segment of the micro-pillar sensor.

Let us first discuss the effect of streamwise pressure gradients ∂*p*/∂*x*. Similarly to the static pressure contribution on the total sensor load in pressure-gradient driven flow, the effect of wall-parallel turbulent pressure fluctuations *p*^′^(*y*) can be discussed. Note, wall-parallel pressure forces *p_P_*(*y*) act in a similar way as drag forces *q_P_*(*y*) on the sensor structure (figure 7(a)). Hence, the ratio of shear load *q_P_*(*y*) and pressure contribution *p_P_*(*y*) allows to estimate the influence of turbulent pressure fluctuations. With equations 2 and 9, *p_P_*(*y*)/*q_P_*(*y*) reads

(11) $$\frac{p_{P}(y)}{q_{P}(y)}\approx \frac{4}{10\pi }\frac{u_{\tau }{}^{2}}{\nu^{2}}\alpha_{2}D_{p}{}^{2}\approx 0.125\frac{u_{\tau }{}^{2}}{\nu^{2}}\alpha_{2}D_{p}{}^{2}$$

It is evident from equation 11 that the effect of horizontal pressure contribution increases with *u*_τ_, i.e., with Reynolds number. Generally valid limits of the Reynolds number, at which the pressure contribution remains negligible, can hardly be given here due to the interdependence of sensor and flow characteristics, but the equations allow to estimate the influence of pressure forces on the total sensor load. At the experimental conditions in [2, 4], the pressure contribution to the sensor load *p_P_*(*y*) is roughly two orders of magnitude below the corresponding shear force per unit length *q_P_*(*y*) such that it can be considered negligible.

#### Wall-Normal Pressure Fluctuations

Note again, that the way, in which wall-normal pressure forces contribute is different from that of wall-parallel fluctuations. The ratio between load contributions resulting from the mean wall-normal pressure gradient and the effect of turbulent wall-normal pressure fluctuations is approximately β_2_^2^*y*^+3^ : β_2_ and hence 10 : 1. That is, wall-normal pressure fluctuations contribute an order of magnitude less to the total pillar bending.

#### Some Conclusion on the Pressure Sensitivity

Let us conclude the above findings. The pressure contribution to the pillar load resulting from wall-parallel and wall-normal pressure gradients have been discussed. These pressure gradients can represent mean pressure gradients in the flow or they might be a consequence/cause of turbulent motion. At the present experimental configurations pressure forces can reliably be neglected. However, with increase in Reynolds number, pressure contributions might represent a non-negligible contribution to the pillar bending. Formula to estimate the influence of pressure gradients have been extensively discussed in this section.

### 6.2. Sensitivity to Cylinder Lift Forces in Shear Flows

It is well known that a sphere [40–43] or a cylinder [44] placed in a shear flow experiences lift forces perpendicular to the mean velocity direction and in the case of the cylinder perpendicular to its axis (figure 7(b)). The integrative effect of this induced lift could add to a deflection of the sensor due to the applying drag forces. However, due to the small lateral dimension of the sensor structure of *D_p_*^+^ < 1, lateral velocity gradients across the sensor diameter, which would cause lift-induced deflections of the sensor structure, can be considered negligible. Note, at scales smaller than the Kolmogorov length scale or viscous length scale, *l_k_* and *l*^+^, respectively, the fluid motion can be considered uniform, and lateral velocity gradients, and hence, lift-force inducing shear-flow conditions, will only arise at larger dimension.

### 6.3. Inertia Sensitivity

Due to its own mass the sensor is sensitive to inertial effects arising from exterior accelerations. Similar to the considerations in the previous section, one possible approach to estimate the sensor sensitivity to acceleration is to relate a deflection caused by inertial forces to a corresponding wall-shear stress, which would have caused the same deflection.

The force *F_inert_*. of a one-g acceleration exerted per unit length of the pillar is *F_inert_*. = ρ*_p_*·*g*·*C_p_*, where ρ*_p_* is the density and *C_p_* the cross section of the pillar sensor. The fluid load per unit length will again be assumed by equation 2. Assuming a constant velocity of *U*(*y*)= *U*_*L*_*p*__ along the sensor geometry, which is good enough as a first rough estimate, and further applying the relation τ*_wall_* = η∂*U*/∂*y* ≈ η*U*_*L*_*p*__/*L_p_*, equation 2 can be reformulated

(12) $$q(y)\approx \frac{4\pi L_{p}}{2-\log_{e}\left(Re_{D_{p}}\right)}\cdot \tau_{\mathrm{wall}}.$$

For Reynolds numbers *Re*_*D*_*p*__ of 10^−3^÷10^0^ equation 12 ranges between

(13) $$q(y)\approx 2/5\cdot \pi L_{p}\tau_{\mathrm{wall}}\ldots 2\cdot \pi L_{p}\tau_{\mathrm{wall}}\approx \pi L_{p}\tau_{\mathrm{wall}}.$$

As such, the equivalent wall-shear stress to a one-g acceleration of the sensor can be expressed by

(14) $$\tau_{\mathrm{wall}}\equiv \frac{\rho_{p}\cdot g\cdot C_{p}}{\pi L_{p}}.$$

At typical pillar dimensions *L_p_* of order 10^−3^ *m* and *D_p_* of order 10^−5^÷10^−4^ *m* and a density of ρ*_p_* ≈ 10^3^ *kg*/*m*^3^ the equivalent shear stress becomes approximately 0.01 *Pa*. Hence, the contribution due to accelerations on the micro-pillar should be accounted for especially if very small wall-shear forces are to be detected. However, if non-accelerating flow facilities and as such non-accelerated micro-pillar sensors are used inertial effects can be neglected. Note, equation 14 is independent of the flow properties, such that the above discussion applies for any flow medium.

### 6.4. Temperature Sensitivity

The sensor material is known to react with an increased shear modulus *G* and Young’s modulus *E* on an increase in temperature [26]. Although the temperature sensitivity of the pillar material has not been investigated in detail some preliminary aspects should be discussed in the following.

Eperiments of micro-pillars in laminar shear flow in a plate-cone rheometer and experiments in turbulent boundary layer flow showed temperature-related problems. In both studies a 100 *W* halogen light source has been used to illuminate the pillars. In air, the large amount of thermal energy, to which the sensor was exposed, led to a heating of the structure.

**Figure 8.**
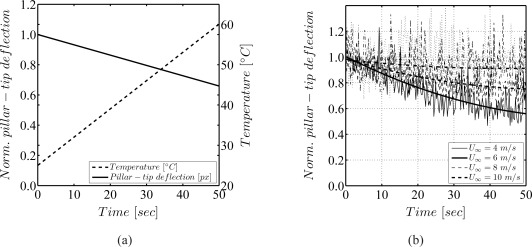
(*a*) Temperature dependence of the pillar-tip deflection in plate-cone rheometer flow at constant rotation. Temperature increase due to illumination. (*b*) Pillar deflection in turbulent boundary layer flow at freestream velocities *U*_∞_ = 4, 6, 8 and 10 *m*/*s*. Pillar deflection has been normalized with the deflection at the beginning of the recording.

Exemplary results evidencing the temperature sensitivity of the sensor are given in 8. Figure 8(a) evidences the sensor deflection to decrease by almost 35% due to an increase of appoximately 35°*C* in laminar rheometer flow. This decrease in the deflection is larger than that predicted by the change in the shear modulus following [26] assuming an inversely proportional relationship between *E* or *G* and the pillar-tip deflection *w*(*L_p_*), which would have yielded deflections of only 15%. However, it was not possible to perform the measurement of the temperature directly at the sensor, which could in fact have been much higher, thereby causing the higher experienced change in the mechanical parameters.

Furthermore, a strong influence of temperature on the elastic behavior of the pillars was observed in turbulent boundary layer air flow measurements (figure 8(b)). The measured values of deflection showed an asymptotic decrease in time at freestream velocities of the turbulent boundary layer flow of *U*_∞_ < 9 *m*/*s*. It is evident from figure 8(b) that increased convection at higher freestream velocities *U*_∞_ reduces the problem. At 10 *m*/*s* the effect evidences to be negligible.

It goes without saying that in water due to the strongly increased thermal convection, the problem of structural heating is less dominant and could indeed not be observed. To completely eliminate thermal effects, it is advisable to use cold-light illumination systems.

In conclusion, it needs to be stated that the sensor evidences to be sensitive to temperature. Therefore, special care has to be taken to minimize systematic errors resulting from thermal effects. To be more precise, the temperature during measurements needs to be kept constant. This, however, is a typical requirement in fluid flow experiments to ensure constant measurement conditions, e.g., a constant Reynolds number. Furthermore, the temperature difference between static and dynamic calibration and measurements should be kept identical.

Assuming a temperature sensitivity (Δ*G*/*G*)/Δ*T* ≈ 0.005/°*C* of the polydimethylsiloxane (PDMS) material given by [27], this leads to an overall uncertainty of the measurement technique due to thermal effects of approximately ±0.25%, assuming the pillar deflection to be more or less inversely proportional to Young’s modulus *E* and the shear modulus *G*.

Thermal expansion effects are, as long as the temperature is kept constant to within ±1°*C*, in the order of 0.1% and can as such be assumed negligible. However, if larger temperature differences are experienced these effects also need to be taken into account.

## 7. Sensor Performance

In the following the sensor performance and design rules for an optimum sensor layout will be discussed. It has become evident that similar to almost all fluid measurement techniques diverse restrictions need to be accomplished for an optimum wall-shear stress measurement with micro-pillars. Since many of these aspects are closely related to the geometry of the flow facilities, e.g., bulk scales, fluid viscosities, etc., no general Reynolds number range can be given here, at which the sensor can be used. Up to now, sensor applications at moderate Reynolds numbers have been successfully performed [4–7]. However, the Reynolds number range in the measurements was rather restricted by the flow facilities and only slight modifications allow the sensor to be used at higher Reynolds numbers.

### 7.1. Sensitivity and Dynamic Range

The sensitivity describes the minimum magnitude of an input signal required to produce a specified output signal. The dynamic range describes the ratio between the smallest and largest possible detectable wall-shear stress. From equation 1 it becomes evident that the magnitude of wall-shear stress depends on the viscosity of the fluid and on the velocity gradient near the wall and it can range from a few *mPa* up to several 100 *kPa*. Depending on whether the flow is laminar or turbulent, the wall-shear stress remains at a constant value or evidences strong fluctuations around this mean value, where the streamwise fluctuation intensity reaches up to 0.4 τ̄*_wall_* [12, 45–48], where τ̄*_wall_* is the mean wall-shear stress. The fluctuations are characterized by a coexistence of a complete spectrum of turbulent structure scales, such that a wall-shear stress sensor needs to be capable of resolving several orders of magnitude of forces. In general, the great range of possible magnitudes of the mean wall-shear stress and of its fluctuations implies that sensors need to be adapted to the flow field, in which they are installed. Due to the great number of partly controversial requirements to be fulfilled by a sensor (see section 2.), it is however almost impossible to have a sensor that covers the complete range of wall-shear stress values. Therefore, normally a sensor is restricted to a certain order of magnitude of shear stress that can be detected. Note, some sensors reported in the literature allow the determination of up to six orders of magnitude [34].

In many applications the value of the wall-shear stress is very low and to increase the sensor’s sensitivity most flow cantilevers and floating-element based sensors cope with the issue of *μ* force measurement by opposing large contact areas to the fluid flow. This automatically leads to a spatial averaging of the wall-shear stress fluctuations over the contact area resulting in a deterioration of the detected dynamic shear-stress characteristics. There still exists a controversial dispute on the maximum allowable sensor length *L* to properly detect the fluctuating velocity or wall-shear stress field in turbulent flows, but a value of approximately *L* ≤ 10÷20 *l*^+^ has been generally accepted sufficient in the literature [48–50].

To explicitly specify the dynamic range of the micro-pillar concept is not an easy task since many aspects such as the sensor sensitivity, the optical resolution, and the quality of the recorded images contribute to the effective dynamic range. Under optimum conditions, the sensor concept has been shown to detect a range of 10^2^÷10^3^ of magnitude of wall-shear stress at a signal-to-noise (SNR) of approximately *SNR* = 10 and more [2, 7].

This implies that the sensor and the optical setup need to be specified in compliance with the shear-stresses present in the flow field. Generally, at each configuration, the maximum detectable shear stress is limited by the endurable mechanical load of the structures. On the other hand, a lower limit is given by the noise of the chosen optical resolution and the image detection and evaluation processes.

Under optimum mechanical conditions, i.e., the sensor bandwidth is not capped by a mechanical overload of the structure, the dynamic range is mostly limited by the image-evaluation routines and is as such comparable to the bandwidth of standard Particle-Image Velocimetry (PIV) [51] and as such approximately two orders of magnitude at a sufficient signal-to-noise level. However, since only single-spot evaluations are performed, and not as in the case of PIV correlations of particle patterns, there is rather no upper limit for the pillar-spot shifts on the recording CCD chip. Allowing shifts of up to 100 *px* increases the possible dynamic range to 10^3^ and more with a remaining SNR of 10 even at the smallest fluctuations.

From equation 2 it is evident that the sensor response is slightly non-linear at small deflections. Furthermore, the sensor structures possess a non-constant diameter *D_p_*(*y*) along the length and a non-negligible curvature at the sensor base. However, as a first rough estimate of the achievable sensor deflections, linear bending theory can be applied assuming a sensor beam with constant *D_p_* with a linear response to the exerting drag forces and the lateral-tip displacement *w*(*L_p_*) can be approximated by

(15) $$w(L_{p})\approx \frac{112}{9}\frac{\tau }{E}\frac{L_{p}{}^{5}}{D_{p}{}^{4}}.$$

Characteristic dimensions and characteristics of sensors used in recent studies are *L_p_* = 350 *μm*, *D_p_* = 45 *μm* and *E* = 1.7×10^6^ *N*/*m*^2^. With values of the wall-shear stress in the range of 10÷1000 *mPa* pillar-tip deflections of 0.1÷10 *μm* can be achieved. Using an appropriately chosen optical resolution of the observing camera system deflections of 101 *px* can be achieved. It goes without saying that field of view decreases at increased optical resolution. Currently available cameras with 1 mega-pixel CCD chips allow to reduce fields of view in the order of 6×6 *mm*^2^ at reasonable pillar deflections in the order of 10^0^÷10^1^ *px*. The use of modern 4 mega-pixel CCDs enables to further increase the field of view at a constant optical resolution.

Note again, that equation 15 should only be treated as a rough estimate and should not be applied to statically calibrate a sensor. The high degree of uncertainty in the exact pillar mechanical parameters, e.g., Young’s modulus, or in the sensor’s geometry, e.g., the diameter *D_p_*, the length *L_p_*, or the base curvature, make a calculation at sufficient accuracy impossible.

### 7.2. Dynamic Response

To detect the complete frequency spectrum of the fluctuating wall-shear stress, a high enough dynamic bandwidth of the sensor structure is required. Depending on the flow characteristics, it can be necessary that the sensor possesses a bandwidth that allows to detect frequencies of a few *kHz*. Furthermore, at increasing frequencies structural scales decrease and the smallest scales, in the following considered to be represented by the Kolmogorov length scale *l_k_*, might range in the order of only a few *μm* depending on the Reynolds number.

It has been shown in [3] that micro-pillar sensors at typical geometries possess eigenfrequencies in the order 400÷2000 *Hz*. Sensors possessing even higher eigenfrequencies of up to 5000 *Hz* have also successfully been manufactured. Note, however, that the increased stiffness of these structures also results in a lower sensitivity (equation 15). The results obtained from an experimental calibration of the sensor structure reported in [3] showed excellent agreement with the findings of a second-order analytical approximation based on experimentally determined damped eigenfrequencies and damping coefficients. The results further yielded the first eigenfrequency *f*_0_ of the structure to be a sufficient parameter to determine the frequency range, at which the sensor possesses a reasonably constant gain. To be more precise, the gain up to approximately 0.3 *f*_0_ was nearly constant.

The transfer function of the structure in water resembles a low-pass filter, i.e., the gain drops at frequencies higher than the damped eigenfrequency *f_D_*. In air, the sensor shows a strong resonance. Therefore, if the sensor is applied in air, it is necessary that the sensor resonance frequency is higher than any expected turbulent frequency, which excites the sensor. In water, the situation is less critical and the sensor can even be applied in flows, in which the turbulent frequencies exceed the dynamic range of the sensor. Within reasonable accuracy, this eigenfrequeny can be analytically approximated by assuming the sensor an (un)damped one-sided clamped beam given by

(16) $$f_{0}\approx \frac{D_{p}}{8\pi }\frac{\lambda_{1}{}^{2}}{L_{p}{}^{2}}\sqrt{\frac{E}{\rho_{p}}.}$$

The quantity λ_1_ = 1.875 is the first eigenvalue for a clamped beam. Note, equation 16 assumes a constant cylinder diameter along the complete length and does not account for non-linear effects at the pillar base. However, the manufacturing process leads to a smooth curvature at the pillar base. A finite-element (FE) eigenfrequency analysis of a simple beam geometry showed typical curvatures at the base in the order of the pillar radius to increase the theoretical eigenfrequencies by 10 ÷ 15%.

That is, again, an exact determination of the sensor properties is impeded by the remaining uncertainty in the determination of characteristic geometric and mechanic parameters of the pillar sensor as has been discussed in [2]. However, for a first definition of the dimension of sensor structure, the above approximation might serve as a useful estimate.

It has already been mentioned that the dynamic response function of the wall-shear stress sensor needs to be chosen in compliance with the Reynolds number and the highest expected frequencies of the investigated flow field. As such, it is necessary to make a rough estimate of the frequency spectrum of turbulent fluctuations existent in the flow at the chosen Reynolds number. The highest characteristic frequencies are related to the smallest-scale structures in turbulent flows. These smallest scales are defined by the Kolmogorov length scale *l_k_* [7, 52, 53]. In turbulent shear flows the ratio between the Kolmogorov length scale *l_k_* and integral scale *l_t_* can be expressed by

(17) lk/lt∼Ret−3/4,

where Ret=(u′2¯)1/2lt/ν is the Reynolds number based on the integral scale *l_t_* and the characteristic velocity of the large-scale eddies represented by the integral scale *l_t_*. The integral scale *l_t_* can be assumed to be approximately 0.1 *δ* [54], where *δ* is the thickness of the shear layer, e.g. in turbulent pipe flow its radius *R* and in channel flow the channel half-height *h*. The eddy velocity can be approximated by the intensity of the velocity fluctuations and is as such ${\left(\bar{{u^{\prime }}^{2}}\right)}^{1/2}=u_{\mathrm{rms}}\approx 0.1\;\;U_{\infty }$ [53], where *U*_∞_ is a characteristics bulk-scale velocity, e.g., the freestream velocity in boundary layers or the bulk velocity in pipe or channel flow. The ratio of the convective time scale (*U*_∞_/*δ*)^−1^ and the Kolmogorov time scale *T_k_* can be expressed as

(18) Tk(U∞/δ)≈Tk(u′2¯)1/2/lt)∼Ret−1/2.

At Reynolds numbers of *Re_b_* ≈ 20000 [4] and characteristic geometric dimensions typical for the measurements performed with the sensor up to now this yields frequencies of the small-scale Kolmogorov structures, i.e., the highest frequencies, to be approximately *f_k_* = 250 *Hz*. The corresponding lengths scales of the smallest structures range in the order of *l_k_* = 60 ÷ 70 *μm*.

Measurements in a turbulent boundary layer flow in air at Reynolds numbers *Re*_Θ_ = 7800÷21000 reveal the general applicability of the sensor technique to such flows [6, 7]. Note, the use in air is comparably more intrinsic compared to that in water. First, as mentioned above, the resonance of the structure causes the sensor to possess a non-constant gain and as such the Reynolds number, at which measurements can be performed, is limited. Furthermore, fluid forces in air are much lower than those in fluids such that the sensitivity of the structure needs to be further increased. This, however, is in contradiction to the aforementioned necessity of a high dynamic bandwidth. The viscous scales in air are smaller compared to those in water, thereby demanding shorter structures, which again competes with the necessary sensitivity. However, applying highly magnifying optics allows to resolve sensor displacements reasonably such that measurements at the aforementioned and even higher Reynolds number are feasible.

### 7.3. Cross-Axis Sensitivity

Cross-axis sensitivity describes the mechanical coupling of perpendicular axis sensitivities. A one-directional sensor device can be sensitive to forces exerted along the axis perpendicular to the axis along which the force is applied. To minimize this kind of cross-axis sensitivity the stiffness of the sensor structure along the perpendicular direction can be chosen much higher than that of the primary axis causing parasitic off-axis contributions to be negligible.

A second kind of cross-axis sensitivity may arise in the case of multi-directional devices where mechanical receptors, e.g., strain gages or piezo-resistive devices, detecting the deflection of sensor elements are sensitive to deflections along perpendicular directions making an identification of the originating force direction difficult or impossible.

Due to its symmetric shape the pillar sensor possesses an identical stiffness along the two perpendicular in-plane directions and is as such a multi-directional sensor at constant sensitivity along all radial directions. Consequently the pillar deflection can be considered a direct representative of the exerted forces, in magnitude and angular orientation. Furthermore, the optical detection principle allows a distinct identification of the two perpendicular wall-shear stress components. That is, a cross-axis sensitivity of the sensor of the second type is not expected.

In consequence of these theoretical considerations, the cross-axis sensitivity has not extensively been studied. However, tests of pillar deflections under varying angular orientations of the sensor in a magnetic field performed in the context of the dynamic calibration described in [3] indicated the cross-axis sensitivity to be indeed negligible.

### 7.4. Repeatability

According to the ‘Guidelines for Evaluating and Expressing the Uncertainty of NIST Measurement Results’ the repeatability of measurement results is defined as the closeness of the agreement between the results of successive measurements of the same measurand carried out under the same conditions of measurement. Repeatability can be expressed as

(19) $$\mathrm{Repeatability}=\frac{\left(\max(w_{i}(L_{p}))-\min(w_{i}(L_{p})))\;\;\;[\mathrm{px}\right]}{\mathrm{FSO}\;\;[\mathrm{px}]},$$

where FSO is the full-scale output. Repeatability tests have been performed under ‘standard’ conditions, i.e., an optical magnification yielding pillar shifts on the recorded images of ≤ 10 *px* has been chosen such that FSO can be considered to be approximately 10 *px*. To reliably investigate the repeatability of the entire sensor measurement chain, i.e., including the pillar deflection, the data acquisition and the image evaluation, measurements need to be performed under well defined experimental conditions. As such, the measurements could not be performed in turbulent flows with statistical fluctuations. Magnetic excitation is used to deflect the pillar from its straight position. The magnitude of deflection depends on the strength of the magnetic field, in which the sensor is placed. The field strength can very precisely be adjusted and kept at a constant level. Repeatability tests at different levels of deflections have been performed and evidenced the repeatability of the sensor to be within ±1.5%*FSO*.

## 8. Conclusion

In this article the Micro-Pillar Shear-Stress Sensor MPS^3^, which offers the potential to measure the two-directional dynamic wall-shear stress distribution in turbulent flows, has been discussed in detail.

The sensor is based on flexible micro-pillars protruding into the near-wall region of turbulent flows and bending in reaction to the exerted drag forces. The deflection of the pillars is detected by optical means and is a representative of the local wall-shear stress. It needs no additional infrastructure on the wall thereby reducing additional flow disturbance such that the pillar technique allows extremely high spatial resolutions of 10^0^ ÷ 10^1^ viscous units and the measurement of the wall-shear stress distribution with up to 1000 sensor posts. It possesses the advantage of very low flow interference. Depending on the geometry and material characteristics of the sensor turbulent scales down to less than 50 *μm* and time scales in the order of a few *kHz* can be resolved making the technology a simple technique to visualize and measure the planar turbulent wall-shear stress distribution of the two wall-shear stress components. Typical micro-pillar sensors possess eigenfrequencies in the order *f*_0_ = 400÷2000 *Hz* and a constant gain up to approximately 0.3 *f*_0_. Sensors with even higher eigenfrequencies of up to 5000 *Hz* have also successfully been manufactured. The sensor concept is reasonably robust and can be easily mounted on almost any surface. Only customary high-speed optics is needed to detect the sensor array.

The present article has discussed in detail material characteristics, possible sensor-structure related errors, various sensitivity and distinct sensor performance aspects. Some guideline to apply micro-pillar sensors to new fields of application has also been given.

The development of the Micro-Pillar Shear-Stress Sensor MPS^3^ can not be considered finished and further improvements will be an exciting challenge for future work. The manufacturing of pillar arrays demands for a better automated positioning of reflective hollow spheres on top of the sensor posts. The implementation of sub-pixel window shifting and adaptive cross-correlation routines will allow for higher achievable accuracy in the detection of the pillar-tip deflection in the order of 0.01 *px*. A concept for a strongly increased magnification of the pillar deflection has been suggested in [7] and would, if applied, allow a further miniaturization of the sensor structures such that pillar sensors could become interesting in the investigation of aerodynamic flows and would further allow the use of the sensor design at higher Reynolds numbers.

## Nomenclature

*D_p_*: Pillar diameter
*E*: Young’s modulus
*f*: Frequency
*f_O_*: Undamped eigenfrequency
*f_D_*: Damped eigenfrequency
*f_k_*: Kolmogorov frequency
*G*: Shear modulus
η: Dynamic fluid viscosity
*l_k_*: Kolmogorov length scale
*l_t_*: Integral length scale
*l*^+^: Viscous length scale (*l*^+^ = *l u*_τ_/ν)
*L_p_*: Pillar length
ν: Kinematic fluid viscosity
*p′*: Pressure fluctuations
*p*: Fluid pressure
*p_P_*(*y*): Pressure load per unit length [*N*/*m*]
*q_P_*(*y*): Shear load per unit length [*N*/*m*]
*Re*_*D*_*p*__: Reynolds number based on a local velocity (e.g., *U*_*L*_*p*__) and the pillar diameter *Dp*
*Re_b_*: Reynolds number based on the bulk velocity *U_b_* and flow facility diameter/height
*Re_t_*: Large-scale turbulent Reynolds number
*Re*_Θ_: Reynolds number based on *U*_∞_ and momentum-loss thickness Θ
*ρ*: Fluid density
*ρ_p_*: Pillar material density
*τ̄*: Mean wall-shear stress
*τ_wall_*: Wall-shear stress
*τ_i_*: Wall-shear stress along the direction *i*
*τ′_i_*: Wall-shear stress fluctuations along the direction *i* (*τ′_i_* = *τ_i_* − *τ̄_i_*)
*u*′,*v*′,*w*′: Velocity fluctuations along the streamwise, wall-normal and spanwise direction
*u*_*τ*_: Friction velocity $\sqrt{\tau_{\mathrm{wall}}/\rho }$
*U*(*y*): Mean local streamwise velocity
*U_b_*: Bulk velocity e.g. in channel/duct/pipe flow
*U*_*L*_*p*__: Velocity at the pillar tip (≡ velocity at the edge of the viscous sublayer)
*U*_∞_: Freestream velocity in boundary layer flow
*w*(*y*): Lateral pillar deflection
*w*_*L*_*p*__: Lateral pillar-tip deflection
*y*: Wall-normal variable/along pillar length
*y*^+^: Non-dimensional (viscous) distance from the wall (*y*^+^ = *u*_τ_*y*/ν)
